# Characterization of tularemia foci in the Republic of Kazakhstan from 2000 to 2020

**DOI:** 10.3389/fepid.2024.1291690

**Published:** 2024-02-19

**Authors:** U. Izbanova, L. Lukhnova, V. Sadovskaya, Z. Zhumadilova, T. Meka-Mechenko, A. Shevtsov, B. Baitursyn, N. Turebekov, N. Tukhanova

**Affiliations:** ^1^ M.Aikimbayev’s National Scientific Center for Especially Dangerous Infections, Almaty, Kazakhstan; ^2^ National Center for Biotechnology, Astana, Kazakhstan

**Keywords:** tularemia, monitoring, epizootic, natural foci, strain, Republic of Kazakhstan

## Abstract

The wide distribution of tularemia in the territory of Kazakhstan is associated with landscape and geographical characteristics. This is explained by a combination of natural factors: the presence of certain types of rodents—reservoirs and sources, ectoparasites—carriers of the causative agent of tularemia. The study of the current spatial and temporal characterization of tularemia in Kazakhstan from 2000 to 2020 will determine the epidemiological status of tularemia and improve the monitoring system in Kazakhstan. In this work we demonstrated the results of a retrospective survey of natural foci of tularemia: analysis of vector, small mammal and human data. The spatial and temporal characteristics of tularemia from 2000 to 2020 in the territory of Kazakhstan were studied in comparison with historical data, including the description of tularemia outbreaks, the clinical picture, and the source of infection, transmission factors, and geographical coordinates of outbreak registration. Sampling was carried out by trapping rodents on snap traps and collecting ticks by rodent combing and by "flagging" methods. For the last 20 years, 85 human cases of tularemia have been reported. During the period from 2000 to 2020, more than 600 strains of *F. tularensis* were isolated from field rodents and ticks in the natural foci of tularemia. MLVA typing of *F. tularensis* strains isolated from natural foci of tularemia in Kazakhstan over the past 20 years. The results of retrospective monitoring indicate that currently active foci of tularemia include the Aktobe, West Kazakhstan, Almaty, East Kazakhstan, and Pavlodar regions. Low-activity natural foci are located in the territory of the Akmola, Karaganda, North Kazakhstan, Kostanay, Atyrau, Zhambyl, and Kyzylorda regions. There are no active natural foci of tularemia in the Mangystau and Turkestan regions. The widespread occurrence of tularemia in the country is associated with landscape and geographical features that contribute to the circulation of the pathogen in the natural focus. An analysis of natural foci of tularemia showed that it is necessary to continue monitoring studies of carriers and vectors for the presence of the causative agent of the *F. tularensis*, in order to prevent mass cases of human disease.

## Introduction

Tularemia is an acute transmissible zoonotic infectious disease caused by the bacterial pathogen *Francisella tularensis*. McCoy and Chapin described for the first time in 1911 a disease comparable to plague in ground squirrels in Tulare County, California. Afterwards, Edward Francis observed that *F. tularensis* was the source of multiple clinical syndromes in humans and suggested the term "tularemia" to characterize the condition (1, 2).

Natural foci of tularemia exist in North America, Europe and most part of Asia (1, 2). The largest number of human cases of tularemia is observed in North America—in the United States of America, in Europe—in Sweden and Finland, and in Asia—in Turkey (3–5). The *F. tularensis* subspecies causing tularemia include subsp. *tularensis* in North America, and subsp. *holarctica* in North America and Eurasia (1, 6). Subsp. mediaasiatica is found in part of Central Asia including Kazakhstan, but has never been isolated from humans.

The first human cases of tularemia in Kazakhstan were identified in 1928 in the settlements located on the Ural River during the harvesting of water vole skins. The disease was accompanied by high fever, enlarged lymph nodes, and ulcers on the surface of the skin. During that period more than 100 patients were registered in a few days (7).

Systematic investigation on tularemia started in 1950 in Kazakhstan. In the following years landscape and geographical conditions, ecological features of the main carriers and vectors of the *F. tularensis*, specific conditions for outbreaks, and seasonality of diseases were characterized. Our researchers have identified natural infestation of *F. tularensis* in 34 species of vertebrate animals and 24 species of invertebrates in Kazakhstan (8).

**Figure 1:**
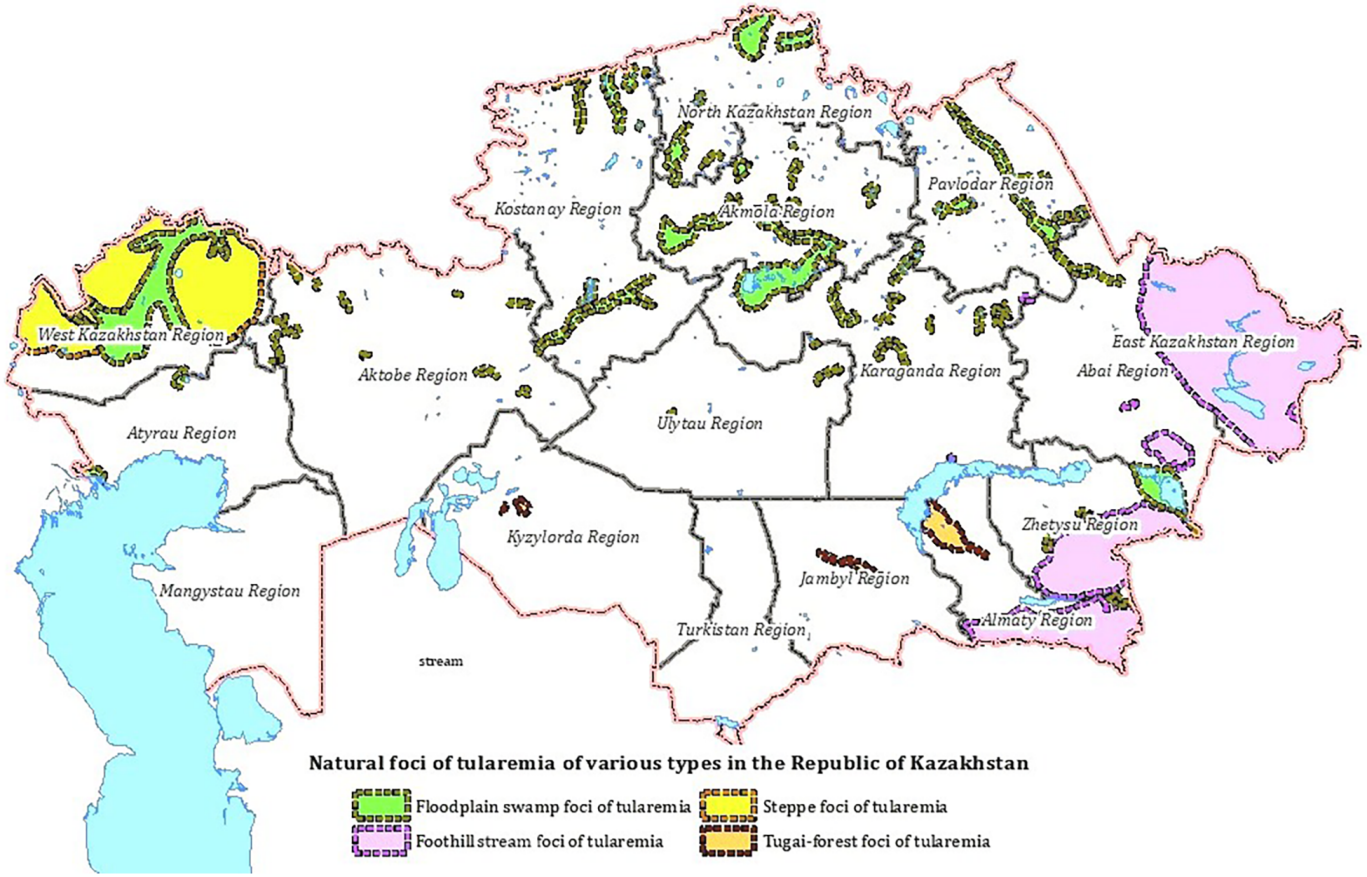
Natural foci of tularemia in Kazakhstan.

A natural focus is a natural ecosystem or biogeocenosis, a component of which is the pathogen population together with the populations of vertebrate hosts that support its existence, and in the case of vector-borne infections, also with arthropod carriers. Four types of natural foci have been identified in the territory of Kazakhstan: floodplain-swamp, foothill-stream, steppe and tugai (Figure 1). Two subspecies of the *F. tularensis* circulate in the territory of Kazakhstan: *F. tularensis* subsp. *holarctica* (biovar I (eryS), biovar II (eryR)) and *F. tularensis* subsp. *mediаasiatica* (7, 8).

Floodplain-swamp foci occupy floodplains of rivers and lake shores, wetlands that are favorable for the habitat of water rats and other moisture-loving animals in the territory of 12 regions of Kazakhstan. The main F.tularensis host is the water vole; the main vectors are ticks of the genus Dermacentor. *F. tularensis* subsp. *holarctica* circulates in the floodplain-swamp type foci (7).

Foothill-stream foci of tularemia have been recorded in the foothills of the Zailiysky, Dzungarian Alatau, Tarbagatai, Altai in the Almaty, Zhetysu, Abai and East Kazakhstan regions. The biocenotic structure is close to the floodplain-swamp type. *F. tularensis* host is the water vole, the main vectors are *Ixodes* and *Gamase* ticks. *F. tularensis* subsp. *holarctica* circulates in this foci (8).

Tugai-type foci are found in the floodplains of rivers in the desert zone, in the Kyzylorda, Zhambyl, and Almaty regions. The main *F. tularensis* carriers are tolai hare, the combed gerbil, and the main vectors are ticks of the genus *Dermacentor*. *F. tularensis* subsp. *mediаasiatica* circulates in the foci (8).

Steppe foci was identified in the West Kazakhstan region at the junction of the river floodplain and the steppe. The main *F. tularensis* carriers are hares and gophers; vectors are ticks of the genus *Ixodes* and *Gamase*. *F. tularensis* subsp. *holarctica* circulates in this focus (8).

In the previous decade human infections were mainly associated with the capture of muskrats, consumption of contaminated water, and tick bites, then since the beginning of 2000, disease have been mainly associated with the consumption of water and food products contaminated by rodents due to their winter migration into human dwellings.

The sporadic incidence and clinical polymorphism of tularemia cause certain difficulties in making a diagnosis, especially in the initial period of the disease. In the early phase of infection, tularemia was often differentiated from acute respiratory infections, pneumonia, tonsillitis, leptospirosis, purulent lymphadenitis, and lymphangitis (9).

In Kazakhstan, two main approaches are used to prevent tularemia cases—vaccination and monitoring of the tularemia in nature. Vaccination of the target population is carried out using live tularemia vaccine strain 15 NIIEG line used in Russia. Routine vaccination of the population with live tularemia vaccine in Kazakhstan began in 1947. Every year, about 12,000 people are vaccinated against tularemia in Kazakhstan as large-scale preventive measures. Routine vaccination is carried out in natural foci of tularemia, where there is a constant circulation of *F. tularensis*, for adults and children from the age of seven. Revaccination is performed every five years (7, 9).

Ecological, zoological, microbiological and molecular biological methods are used to monitor the circulation of tularemia in natural foci. The isolation and genotyping of the pathogen, followed by its mapping in the field, makes it possible to assess and predict the spread of infection.

Currently, MLVA is recognized as the most discriminatory genotyping method based on VNTR. The method provides sufficient information for epidemiological monitoring and determining the geographical origin of infection (10).

The data obtained will make it possible to create a map of the distribution of *F. tularensis* genotypes in the territory of Kazakhstan and will be used in epidemiological monitoring of subsequent outbreaks. Publication of data and replenishment of international MLVA databases of *F. tularensis* genotypes circulating in Kazakhstan will be relevant for world science in the global control of tularemia.

The aim of this study is to characterize the spatial and temporal structure of natural foci of tularemia, to assess the epizootic and epidemic situation in the period from 2000 to 2020 in Kazakhstan.

## Materials and methods

### Study area

Kazakhstan includes 17 administrative regions, with a total area of 2,724,902 km^2^. The length of the country east—west is greater than south—north. Every year, as part of tularemia monitoring, the territories of 2,740 settlements were surveyed, with an area of 510,000 km^2^. In this work we used the results of epizootiological examination of natural foci of tularemia based on official information of anti-plague stations, and departments of sanitary and epidemiological control of the regions. Data on the incidence of tularemia for 20 years were analyzed from official sources of the sanitary and epidemiological service of the Republic of Kazakhstan (11).

### Sample collection

In the period from 2000 to 2020, the collection of biological material in natural foci of tularemia from rodents and ectoparasites was carried out by specialists from branches of our center as part of annual activities aimed at controlling tularemia in Kazakhstan. About 20,000 rodent specimens and about 220,000 ticks are collected to monitor *F. tularensis* territories of natural foci during 2000–2020 in Kazakhstan.

Small mammals were captured using traps of different sizes, set overnight at 10 m intervals, and baited with bread or cured pork fat. Ticks were collected from rodents and other small mammals using the scratching method. Small mammals were identified at the species level by experienced zoologists, then necropsy was performed, and internal organs (liver, spleen) aseptically collected.

The collection of ticks in the field was carried out using a “Flagging” method. Collected ticks were identified by species based on morphological characteristics following the official guidelines for tick species identification in Kazakhstan (12, 13). Ticks were grouped into pools by species, life stage and sex (with a maximum of 5 adult ticks in a pool). Internal organs of small mammals and collected ticks homogenized and studied by bacteriological, serological, and molecular methods. The identification of *F. tularensis* strains was carried out using generally accepted laboratory diagnostic methods (14).

### Genomic analysis

DNA samples isolated from *F. tularensis* strains were used for MLVA genotyping. The strains were isolated in the territory of natural foci of tularemia in Kazakhstan for the last 20 years. Isolates were isolated from the following animal species: *Mus musculus, Meriones tamariscinus, Apodemus uralensis, Arvicola amphibius, Crocidura suaveolens, Meriones meridianus, Microtus arvalis, Mustela nivalis, Spermophilus pygmaeus, Allactaga major, Cricetulus migratorius, Oenanthe isabellina, Spermophilus major*. These strains were isolated during routine epizootiological investigations of natural foci of tularemia. DNA was isolated using the QIAamp DNA Mini Kit (Qiagen, Germany).

MLVA genotyping. 10 VNTR loci were included in the MLVA genotyping panel Ft-M2, Ft-M3, Ft-M4, Ft-M5, Ft-M6, Ft-M10, Ft-M20, Ft-M22, Ft-M23, and Ft-M24. The loci were amplified according to the protocol proposed by A J Vogler et al. (15) (The FT-20-2B primers were not used due to the invariance of this region of the Ft-M20 locus in *F. tularensis* subsp. *holarctica*). PCR products were separated by capillary electrophoresis on a 3730xl DNA Analyzer in the presence of the Liz1200 size standard. Allele size was identified in GeneMapper 4.1 software (Applied Biosystems). Strains with a complete genome available and known MLVA profiles were used as controls (16). To visualize clustering relations, maximum parsimony analysis dendrograms were constructed using BioNumerics 8 (Applied Maths, Sint-Martens-Latem, Belgium). The Simpson's Discrimination Index was used to evaluate each VNTR locus and their total discrimination (17).

## Results

We have conducted a study of epidemiological monitoring of natural foci of tularemia in Kazakhstan in the period from 2000 to 2020, in comparison with historical data. During this period 85 human cases were officially registered. The largest number of diseases was registered in the floodplain-swamp foci—51 cases, in the foothill-stream foci—30 cases, and in the steppe—4. The disease occurred mainly through the bite of ticks and horseflies, or was associated with the consumption of water and food products contaminated by rodents. Glandular and oropharyngeal forms of the disease predominated in the patients. Most infections were of mild to moderate severity and occurred in people of working age (88.5%). In terms of gender composition, men predominate among the adult population—74%.

In the West Kazakhstan region there are floodplain-swamp and steppe foci of tularemia. The steppe foci cover the northern and central regions of the region (18). The floodplain-swamp focus occupies large areas of five districts. In these foci, 16 species of mammals are involved in the epizootic process mainly water voles, hare, small ground squirrel, house and wood mice, common and gray hamster, common vole, steppe pied. The main vectors are *Ixodes* ticks of the species *Dermacentor marginatus, Dermacentor pictus, Rhipicephalus pumilio, Rhipicephalus rosicus*. Large epizootics of tularemia among rodents were noted in 1955–1957 and were associated with the mass reproduction of steppe lemmings. From 2011 to 2013, tularemia epizootics were recorded involving house mice, comb and midday gerbils, shrews, small and large ground squirrels. For the period from 2000 to 2020, epizootic activity in the region is high, strains of the *F. tularensis* are isolated annually, and specific antibodies are detected in rodents.

At the beginning of the 20th century, before mass vaccination, a high level of tularemia epidemic activity was noted in the West Kazakhstan region. The first human case of tularemia was registered in 1928 in the West Kazakhstan region and all cases were associated with the harvesting of water vole skins (19). From 1943 to 1965, about 50 outbreaks of tularemia were observed in the region (8, 18). The high level of vaccination against tularemia has reduced the incidence of the disease to single cases. The last cases of tularemia in the natural foci of the West Kazakhstan region were registered in 2002 and in 2007.

In the territory of the North Kazakhstan region there are active natural tularemia foci of the floodplain-swamp type with a high risk of disease cases. The main source in these foci is the water vole. The study of spatiotemporal characteristics of tularemia showed high intensity of epizootics from 1945 to 1999. Between 2000 and 2020, research on rodents and mammals found only a few samples positive for *F. tularensis* antibodies and antigens, but no strains of this pathogen were isolated. The first human case of tularemia in the North Kazakhstan region was registered in 1945. More than 300 cases were reported between 1949 and 1972 (20). During the period from 2000 to 2020, 11 human cases were registered and most of them were related to gadfly bites, consumption of water and food infected with excretions of rodents and the glandular form of tularemia was predominant.

In the Pavlodar region, floodplain-swamp foci and one steppe foci of tularemia are located in the territory of eleven districts. The study of the current spatial and temporal characterization of tularemia in Pavlodar showed high intensity of tularemia epizootics from 1953 to 1972. For the last 40 years, no human cases have been recorded, although *F. tularensis*-positive samples (antibodies or antigens) were detected in rodents and ectoparasites during this period (21). Human cases were reported in 2002, 2003 and in 2016, 2017. In the period from 2010 to 2020, about 200 strains of *F. tularensis* were isolated in the territory of the Pavlodar region. The natural foci of tularemia existing in this region may cause sporadic cases among the population.

Natural foci of tularemia of the Kostanay region are located in the territory of eight districts. The water vole is the main carrier of *F. tularensis* in this area. The common vole, steppe lemming, wood mouse and house mouse are also involved in the epizootic (8). The main vectors are *Ixodes* ticks of the species *D. marginatus*, *D. pictus*. In the period from 1973 to 1991, epizootics among rodents were registered annually. From 1943 to 1962, about 70 human cases of tularemia were reported. Single cases have been reported in 1973, 1993, and in 2018. From 2000 to 2020 there was no isolation of *F. tularensis* from ectoparasites and rodents. Currently, the natural foci are not active.

In the territory of the Zhambyl region the first natural foci of tularemia was detected in 1941. There are two natural foci of tularemia including tugai focus-in one district and a foothill-stream focus in two districts (22). In the period from 1969 to 1998, spillover epizootics of tularemia among rodents were registered annually. In 2003 and 2004, few *F. tularensis* strains were isolated, mainly from *D. niveus* ticks. Human cases of tularemia were registered in 1941 and in 1968, as a result of tick bites. Analysis of the tularemia situation in the territory of the Zhambyl region showed that the tugai foci did not present high epizootic activity.

In the territory of the Almaty region there are two foci of tularemia of the foothill-stream type, four foci of the floodplain-swamp type, one focus of the tugai type. The foci are located in 16 districts. The Zaili foothill-stream focus is inactive, and the activity in the Dzungar foothill-stream focus of tularemia is reduced (23). From 1997 to 2016, single human cases were reported, and from 2017 to 2020 human cases were not reported. The epizootic activity of floodplain-swamp foci (Karatal, Alakol, Lepsin) is low. For the last 15 years, strains of the *F. tularensis* have not been isolated. The focus of the tugai type in the territory of the Almaty region is weakly active throughout the entire observation period. Since the discovery of the focus of the tugai type, no more than 20 strains of the *F. tularensis* have been isolated; several cases of human disease have been registered in 1965 and in 1968. A total of eight cases of human disease have been registered in the period from 2000 to 2020 in the territory of the Almaty region, the focus is active.

In the territory of the East Kazakhstan region there are four natural foci of tularemia; two of them are foothill-stream type and two are floodplain-swamp type. The presence of tularemia in the Alakol floodplain-swamp focus was established in 1959. More than 1000 patients were reported in the East Kazakhstan region between 1942 and 1962. Until 2000, single human cases were recorded; from 2000 to 2020 more than 40 human cases were registered (23, 24). Currently investigation of rodents, ectoparasites, and environmental samples showed a low percentage of positivity for tularemia. At present epizootic activity persists in floodplain-swamp and foothill-stream foci.

The territory of the Atyrau region includes floodplain-swamp foci occupying numerous arms of the Volga river and the coastal part of the Caspian Sea. Monitoring data indicate tularemia cases were detected among rodents and humans until 1965 (8). In the following years until now epizootic and epidemic data on tularemia show that the Atyrau region is inactive.

In the territory of the Karaganda region, there are two types of tularemia foci—floodplain-swamp and foothill-stream. The floodplain-swamp foci of tularemia are located in the territory of four districts, the foothill-stream foci are located in the territory of the two districts (8). The results of a retrospective study of the current spatio-temporal characteristics of tularemia from 2000 to 2020 indicate that the epizootic activity of floodplain-swamp and foothill-stream tularemia foci has been reduced in the Karaganda region, and human tularemia cases have not been reported for more than 50 years. There are only rare cases of positive serological findings in the study of rodents and ectoparasites.

In the Akmola region there are floodplain-swamp type foci located in seven districts. The results of retrospective analysis indicate that the epizootic activity of tularemia floodplain-swamp foci in the region has been reduced. Since 2013, no strains of *F. tularensis* have been isolated, only rare cases of positive serologic findings in rodents and ectoparasites have been noted. In 2001, 2005, 2012, human cases were reported.

In the territory of the Aktobe region there are two floodplain-swamp foci type. The aggravation of the epizootic process in the territory was noted in 1980 and 1986 (8, 9). In the period from 2003 to 2020, a continuous activity is recorded, a high activity of the natural foci is noted, which is confirmed by the isolation of strains from *Ixodes* ticks and rodents. Activity of tularemia is registered annually; natural foci remain epizootically active. In the Aktobe region in 1984, in 2003 and in 2007 human cases of tularemia were registered, and infection was associated with rodents.

In the Kyzylorda region in the lower reaches of the river Syrdarya there is a tugai type of tularemia focus. In the period from 1957 to 1965, the tugai focus was active; the last human case of tularemia was registered in 1958 (8, 9). Currently, this region is not active. Long term examination indicates the absence of epizootic activity in this region.

In the Mangistau and Turkestan regions, no cases of human disease and no epizootics of tularemia among rodents have been registered.

The results of molecular analyzes of 10 VNTR loci were obtained for all strains (Supplementary Table S1). The FT-M02, FT-M10, and FT-M23 loci were identical among the analyzed strains of *F. tularensis subsp. holarctica* (*n *= 143) (Table 1). The highest discrimination ability was noted in the FT-M03 locus, in which 16 alleles were identified, and the discrimination index was 0.9052. Four alleles were identified at the FT-M6 locus with a discrimination power of 0.6761. At loci FT-M04 and FT-M20-A, FT-M22, FT-M24, 3 and 2 alleles, respectively, were identified, and the discrimination index varied from 0.1430 to 0.0278. The discrimination index for all 10 loci was 0.9256.

**Table 1:** Simpson's diversity index value of variable number tandem repeat (VNTR) loci, D.

Locus	Allele number	Simpson's diversity index (total *n *=* *143)	Standard deviation
FT-M02	1	0.0000	[0.0000, 0.0000]
FT-M03	16	0.9052	[0.8837, 0.9268]
FT-M04	3	0.1326	[0.0573, 0.2079]
FT-M05	1	0.0000	[0.0000, 0.0000]
FT-M6	4	0.6761	[0.6487, 0.7034]
FT-M10	1	0.0000	[0.0000, 0.0000]
FT-M20-2A	2	0.0414	[0.0000, 0.0873]
FT-M22	2	0.1430	[0.0676, 0.2184]
FT-M23	1	0.0000	[0.0000, 0.0000]
FT-M24	2	0.0278	[0.0000, 0.0660]
MLVA10	28	0.9256	[0.9020, 0.9492]

**Figure 2:**
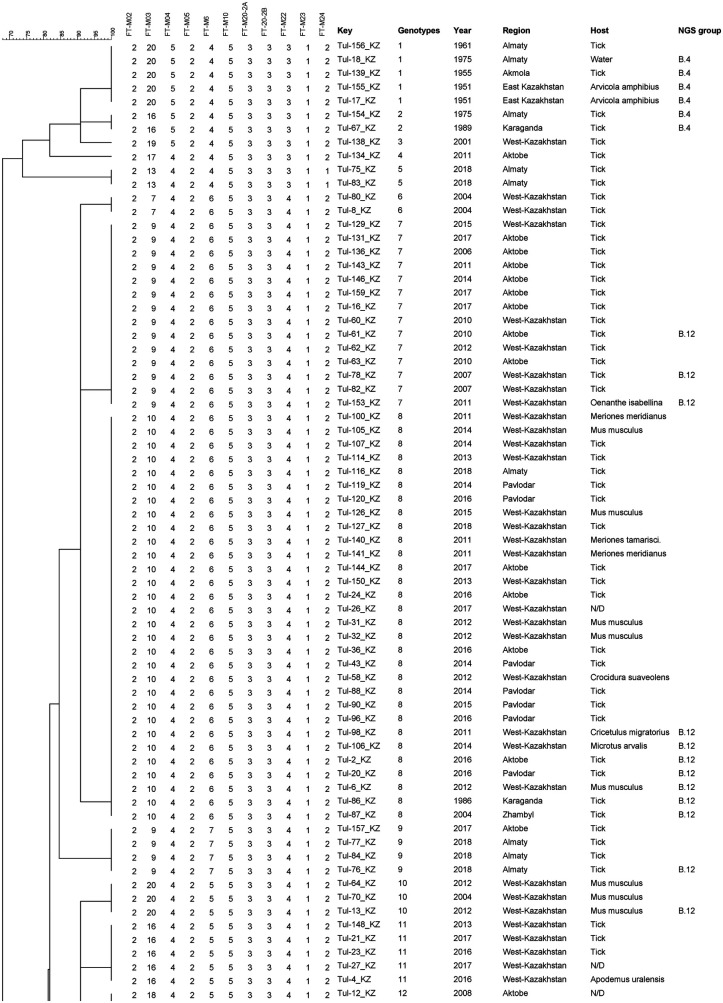

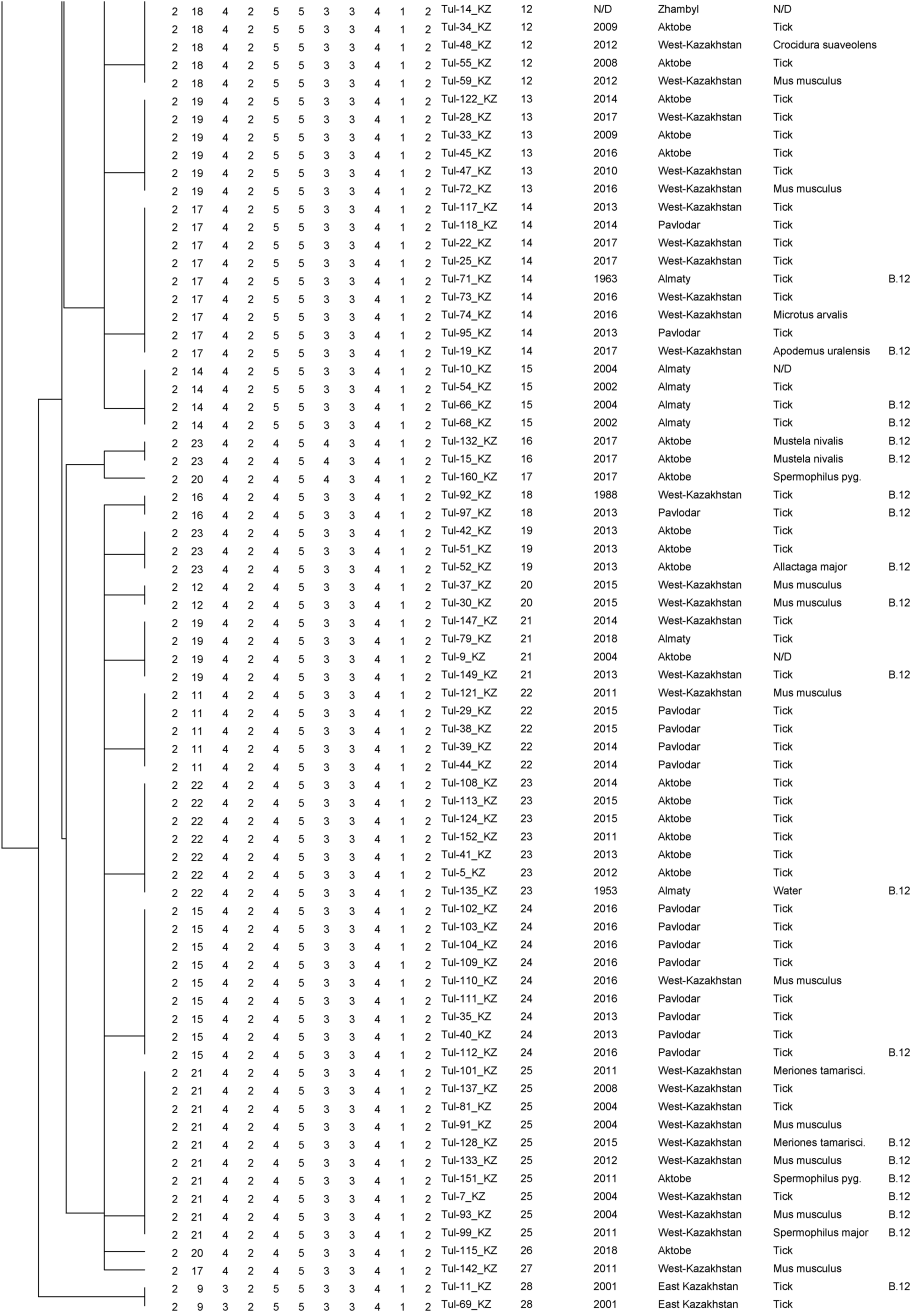
Fragment of the phylogenetic tree of tularemia microbe strains on the basis of 25 VNTR markers.

The analyzed samples (*n *=* *143) were clustered into 28 genotypes, which were divided into two clusters (Figure 2). The first cluster included 11 strains that formed 5 genotypes, of which 6 strains were assigned to the B.4 line based on WGS. The second cluster united the remaining 132 strains clustered into 23 genotypes, of which 15 genotypes include full genome strains assigned to the B.12 lineage. The first and second cluster, based on the distribution of strains with WGS, can be considered as B.4 and B.12 lines, which differ at the FT-M22 locus.

The first cluster included strains isolated in 6 regions of Kazakhstan in 1951–2018. The largest genotype combined 5 strains from 3 regions of Kazakhstan between 1951 and 1975. Two genotypes are combined by 2 strains and 2 genotypes are represented by one strain. Of the 23 genotypes of the second cluster, 3 genotypes represent individual strains, 5 genotypes 2 strains each, of which 4 genotypes combine strains isolated from one region and one year of isolation, and 18 genotypes combine strains from two regions isolated in 1988 and 2013. Among the genotypes, including 3 or more strains, only 4 genotypes are represented by strains from the same region with a difference in isolation within the genotype of a maximum of 8 years. The largest genotype 8 combined 30 strains isolated from 6 regions in the period from 1986 to 2018. The remaining genotypes also combine strains from different regions thousands of kilometers apart.

## Discussion

The spatiotemporal data of this study for the period 2000 to 2020 indicate that there are still active foci of tularemia in Kazakhstan. Currently, active foci of tularemia are the Almaty, Aktobe, West Kazakhstan, East Kazakhstan, and Pavlodar regions. The epizootic and epidemic risk in tularemia natural foci of these regions is high. There is also a high risk of occurrence of sporadic tularemia cases in the human population. Under favorable conditions, an increase in the number of rodents or ectoparasites could transform a local epizootic process into a diffuse epizootic. Comprehensive monitoring of the state of natural foci of tularemia includes monitoring of natural strains circulating in the territory. In the territory of Kazakhstan, *F. tularensis* subsp. *holarctica* and subsp. *mediasiatica* strains circulate (7, 9). As for subsp. *holarctica*, there are both erythromycin susceptible (Ery-S, biovar 1) and erythromycin resistant (Ery-R, biovar 2) strains. The strains isolated in 2000–2020 showed the stability of traits typical for the *F. tularensis* of the Holarctic and Central Asian subspecies.

At the present time, tularemia is characterized by sporadic cases in Kazakhstan. The largest number of disease was registered in the floodplain-swamp foci of tularemia. The disease occurred mainly from tick bites and consumption of water and food products infected with rodents, which prevailed among individuals not vaccinated against tularemia. In many countries, the main routes of human infection with tularemia remain contact with contaminated water and animal excrement in food storage areas (4, 25).

Routine vaccination against tularemia contributed to the formation of a highly immune layer among the population living in the territory of natural foci of tularemia and restrained the spread of infection, despite the high epidemic potential of tularemia foci. The stability of the incidence over a number of years, at a relatively low level, indicates fairly effective anti-epidemic measures against tularemia.

Currently, active foci of tularemia are the Almaty, Aktobe, West-Kazakhstan, East-Kazakhstan, North-Kazakhstan, and Pavlodar regions, where tularemia pathogens are annually isolated from rodents and ectoparasites, as well as registered human cases.

According to the results of the study, it was determined that the epizootic and epidemic potential of tularemia foci in the territory of the Akmola, Kostanay, Atyrau, Zhambyl, Kyzylorda, and Karaganda regions has been reduced. In these areas, the number of rodents and ectoparasites is reduced, the causative agent of tularemia is not isolated, and human diseases are not recorded. It was determined that in the Atyrau region there are no conditions for the existence of the Inder floodplain-swamp focus; anthropogenic transformations have changed the structure and activity of the focus. Many natural phenomena (winter frosts, spring floods, drought, natural fires) affected the number of mouse-like rodents and water voles. Activation of the focus is possible with favorable climate change, an increase in the number of rodents and ectoparasites.

MLVA genotyping was carried out according to a genotyping scheme that included 25 loci (6). MLVA analysis clustered the studied *F. tularensis* subsp. *holarctica* strains into 28 genotypes, which were divided into two clusters. In our study, six strains isolated in 1951–1989 in Central and Eastern Kazakhstan were assigned to the genetic group B.4. Strains belonging to the B.4 genetic group have previously been detected in North America, China and Northeastern Europe (2, 6, 26). The B.4 genotype may be of Asian origin and is mainly distributed in Asia. The remaining strains from Kazakhstan were assigned to the genetic group B.12, which is widespread in Central, Eastern and Western Europe (27–29).

The epizootic process in a natural focus of tularemia consists of two factors: the persistence of the pathogen during the inter-epidemic period and the circulation of the pathogen during the epidemic period (25, 30).

During the inter-epidemic period, the pathogen remains in the environment in an inactive state, while retaining the ability to revert into vegetative virulent forms when living conditions change (30, 31). This explains why the genotypes of *F. tularensis* present in a certain natural focus of tularemia can persist there for several decades (30).

The increase in the incidence of human cases of tularemia could occur in the event of an increase in the frequency of epizootics among highly susceptible small mammals or in the number of arthropod vectors (4, 31). Despite the low epizootic activity of tularemia foci in some areas, it is necessary to continue monitoring studies, increase the coverage of settlements with zoological and parasitological work, and take timely preventive measures, including vaccination of the population.

## Conclusions

Continuous monitoring of natural foci of tularemia, particularly in the animal and arthropod reservoir, is necessary. Priority actions to combat tularemia, depending on epizootiological and epidemiological data, must be implemented to control this infectious risk in Kazakhstan.

The genomic characteristics of *F. tularensis* strains isolated in Kazakhstan were studied using multilocus analysis; 28 genotypes and two clusters were identified.

The manifestation of the epizootic activity of the natural focus of tularemia in some cases becomes known after the registration of a human disease. A change in indicators and predictors towards an increase in diseases and another will indicate a worsening of the epizootic and epidemic situation for tularemia. The deterioration of the epidemiological situation of tularemia is, as a rule, a consequence of a decrease in the volume of preventive work, a reduction in the area of epizootiological examination of natural foci, and a reduction in the coverage of vaccinations against tularemia of the population. It is necessary to carry out continuous comprehensive monitoring of the territory of natural foci of tularemia with mandatory molecular genetic studies of strains.

## Data Availability

The original contributions presented in the study are included in the article/Supplementary Materials, further inquiries can be directed to the corresponding author.
